# Copper-Integrated Aminated/Amidine-Functionalized Acrylic Textile for High-Stability HRP Immobilization and Bisphenol A Removal

**DOI:** 10.3390/polym18111364

**Published:** 2026-05-31

**Authors:** J. Alkabli, Naif Abdullah R. Almalki, Yaaser Q. Almulaiky

**Affiliations:** 1 Department of Chemistry, College of Science, University of Jeddah, Jeddah 23218, Saudi Arabia; 2 Department of Biochemistry, King Abdulaziz University, Jeddah 21589, Saudi Arabia; naralmalki@kau.edu.sa; 3 The Applied College, University of Jeddah, Jeddah 23218, Saudi Arabia

**Keywords:** textile-based biocatalyst, aminated/amidine, Bisphenol A degradation, immobilization

## Abstract

This work introduces a textile-based platform for biocatalysis by integrating a copper-based hybrid domain onto aminated/amidine-functionalized acrylic textile (TAC–Cu), producing a functional bio-textile capable of high-performance enzyme immobilization. The textile substrate was chemically modified with ethylenediamine to generate amine/amidine-type functional groups, enabling in situ formation of copper-based hybrid structures through either a conventional solvothermal approach or a plant-mediated route employing *Costus speciosus* extract. The green-synthesized TAC–Cu composite exhibited superior structural uniformity, improved porosity, and enhanced surface chemistry, resulting in a higher horseradish peroxidase (HRP) immobilization yield (92%) compared with the chemically synthesized analogue. The resulting HRP-functionalized bio-textile demonstrated markedly improved catalytic behavior, including a reaction rate constant nearly twice that of the free enzyme, and strong operational robustness. As a technical textile engineered for environmental applications, the composite achieved 90% bisphenol A (BPA) removal within 90 min and retained substantial enzymatic activity even at 80 °C, whereas free HRP was almost fully deactivated. Overall, this study highlights the potential of eco-engineered TAC–Cu materials as a new class of functional and sustainable bio-textiles, combining enzyme stabilization, high catalytic efficiency, and suitability for wastewater treatment and other technical textile applications.

## 1. Introduction

Porous metal-based hybrid materials (PMBHMs) have attracted increasing interest in recent years because of their high surface area, tunable structural features, and versatile coordination chemistry, which make them promising candidates for adsorption, catalysis, biocatalysis, and environmental remediation [1,2,3]. In recent years, PMBHMs have also emerged as promising platforms for enzyme immobilization because their porous structures, accessible active sites, and tailorable surface functionalities can provide favorable microenvironments for enzyme anchoring and catalytic preservation. Enzyme immobilization has become an important strategy for overcoming the intrinsic drawbacks of free enzymes, such as poor operational stability, low resistance to pH and temperature fluctuations, difficult recovery from reaction media, and limited reusability. By attaching enzymes onto or within suitable carriers, immobilization can improve enzyme handling, facilitate separation, enable repeated use, and often enhance structural stability during storage and operation. Despite these advantages, immobilization may also introduce certain limitations. Depending on the support material and immobilization pathway, the catalytic efficiency of the enzyme may decrease because of steric hindrance, conformational changes, diffusional resistance, or reduced accessibility of substrates to the active site. In addition, weakly adsorbed enzymes may leach from the support during repeated cycles, while dense or poorly designed matrices may limit mass transfer. Therefore, the performance of an immobilized enzyme system strongly depends on the nature of the carrier, the strength of enzyme–support interactions, the accessibility of the catalytic interface, and the preservation of the native enzyme structure. For this reason, extensive efforts have been devoted to developing advanced immobilization supports, including inorganic oxides, carbon-based nanomaterials, polymeric matrices, magnetic particles, porous hybrids, and coordination-based materials [4,5].

Traditionally, many coordination-based porous materials are synthesized through solvothermal or hydrothermal methods that require high temperatures, organic solvents, and lengthy reaction times, often resulting in environmental concerns and limited scalability [6]. In response, researchers have increasingly explored green synthesis strategies, incorporating plant extracts as benign reducing, stabilizing, and structure-directing agents. Such biogenic approaches offer low toxicity, cost-effectiveness, and environmental compatibility [7,8,9]. Furthermore, several studies have reported the successful use of extracts from *Aloe vera*, *Azadirachta indica*, *Ocimum basilicum*, and *Vitis vinifera* in the synthesis of metal nanoparticles or hybrid composites [10,11,12,13]; however, their role in constructing coordination-based porous architectures remains relatively underexplored. This gap is particularly significant for enzyme immobilization applications, where green fabrication strategies may provide notable advantages in terms of biocompatibility, milder synthesis conditions, and reduced exposure to enzyme-denaturing chemical residues. Meanwhile, substantial efforts have been directed toward the development of polymer–coordination hybrid materials and the integration of coordination-based porous coatings onto flexible substrates such as cellulose fibers, chitosan, and synthetic polymers to improve mechanical stability and support practical application [14,15,16,17]. These hybrid materials are particularly attractive for immobilized biocatalyst design because they combine the structural and chemical merits of coordination-based porous materials with the mechanical flexibility, processability, and easy recoverability of polymeric supports. Flexible fibrous substrates can further reduce particle aggregation, facilitate handling, and provide more accessible surfaces for enzyme loading. Despite these advantages, acrylic fabrics, as widely available and low-cost synthetic polymers, have received relatively little attention as scaffolds for coordination-based porous coating growth. Moreover, although several functionalization strategies such as plasma treatment and graft polymerization have been reported [18], the use of ethylenediamine to chemically modify acrylic fabric and introduce amine-rich reactive sites remains underexplored, particularly as a platform for metal coordination, porous network formation, and subsequent enzyme immobilization. Importantly, *Costus speciosus*, a medicinal plant rich in flavonoids, saponins, and alkaloids, has not yet been explored for the synthesis of coordination-based porous materials or related copper-based hybrid systems. The phytochemical richness of this extract offers unexplored potential for directing network formation, improving particle dispersion, and enhancing biocompatibility. These properties may be especially beneficial in enzyme-support design, where interfacial chemistry and microenvironmental compatibility are crucial for preserving catalytic activity after immobilization. To the best of our knowledge, no comprehensive study has yet combined acrylic textile functionalization with plant-mediated formation of copper-based hybrid layers using *Costus speciosus* extract, nor systematically compared plant-mediated and chemically synthesized counterparts to elucidate the role of the biogenic component. In this study, we address this gap by developing a novel two-step fabrication strategy. First, acrylic fabric was chemically modified through reflux treatment with ethylenediamine to generate a reactive aminated/amidine functionalized structure (TAC). Second, the functionalized fabric served as a support for the formation of copper-based hybrid domains through a solvothermal route, either with or without *Costus* speciosus extract as a biogenic mediator. The resulting materials, plant-mediated TAC-Cu and chemically synthesized TAC-Cu, were characterized and compared to clarify the influence of the biogenic component on structural development and surface morphology. The optimized hybrid support was then utilized for HRP immobilization to construct a reusable and efficient biocatalytic system. Overall, this work presents a hybrid biocatalytic platform integrating polymer surface functionalization, plant-mediated copper incorporation, and enzyme immobilization, offering promising potential for catalytic and environmental applications.

## 2. Materials and Methods

The acrylic textile fabric used in this study was supplied by Misr El-Mahalla Co., El-Mahalla El-Kubra, Egypt. The fabric was woven in a plain weave (1/1) structure with a thread count of 40.6 threads per inch (TPI) in both the warp and weft directions and a density of 0.36 g/cm^3^. Commercial acrylic fibers are generally based on polyacrylonitrile (PAN) copolymers containing a high proportion of acrylonitrile units together with minor amounts of comonomers such as acrylate or methacrylate derivatives introduced to improve flexibility, dyeability, and processing behavior. Consequently, the pristine acrylic fabric contains abundant nitrile (–C≡N) groups that serve as reactive sites for ethylenediamine-mediated functionalization, while ester/carbonyl-containing comonomer units are also present as part of the commercial acrylic copolymer structure. Key biochemical reagents, including bovine serum albumin (BSA), horseradish peroxidase (lyophilized powder, activity ~150 U/mg), guaiacol, hydrogen peroxide, ethylenediamine (99% purity), sodium carbonate, and copper (II) nitrate trihydrate (Cu(NO_3_)_2_·3H_2_O), were obtained from Sigma-Aldrich Ltd. (St. Louis, MO, USA). All solvents (ethanol, isopropanol, acetonitrile, dioxane, and acetone) were of analytical grade and supplied by Merck Ltd. (Budapest, Hungary).

### 2.1. Preparation of Costus Extract

To prepare the *Costus* extract, dried roots of *Saussurea costus*, obtained from a local herbal market in Jeddah, Saudi Arabia, were first ground into a fine powder. Aqueous extraction was performed by heating 2 g of the powdered root in 20 mL of distilled water at 70 °C for 20 min. The resulting mixture was then centrifuged at 6000× *g* to separate the supernatant. The obtained *Costus* extract was used in the synthesis process as a green, biocompatible agent serving both reducing and stabilizing functions. Previous green chemistry studies have highlighted the effectiveness of *Costus*-derived phytochemicals in facilitating metal oxide formation and enhancing enzyme stabilization [19].

### 2.2. Synthesis of Chemically and Plant-Based Copper Integrated Textile Hybrids (TAC–Cu)

Acrylic fabric (3 g) was first thoroughly washed with distilled water and air-dried at room temperature. It was then immersed in 40 mL of dioxane and gently stirred before the addition of 60 mL of ethylenediamine (99%) and 0.5 g of sodium carbonate. The mixture was subjected to a reflux reaction in a paraffin oil bath at 110 °C for 12 h. The resulting modified fabric (TAC) was filtered, washed several times with distilled water, rinsed with acetone, and air-dried to obtain a reactive powder.

For the synthesis of the copper-based textile hybrids (TAC–Cu), the TAC powder was dispersed in an ethanol–water mixture (60:40, *v*/*v*) and combined with 10 mmol of copper nitrate. After stirring and preheating at 80 °C for 30 min, the suspension was divided into two portions. The first portion underwent solvothermal treatment in a Teflon-lined stainless-steel autoclave at 80 °C for 8 h. The resulting product was centrifuged, washed with distilled water and acetone, and dried at 90 °C to yield the chemically based TAC–Cu material. The second portion was supplemented with 20 mL of *Costus* extract and heated for an additional 30 min at 80 °C before undergoing the same solvothermal treatment. After cooling, centrifugation, washing, and drying, the resulting product was labelled as the plant-based TAC–Cu material.

### 2.3. HRP Immobilization

To immobilize horseradish peroxidase (HRP), 0.5 g of the TAC–Cu composites was first activated by incubating it in a 1% glutaraldehyde solution prepared in 0.1 M sodium phosphate buffer at pH 7.0. This suspension was stirred at 30 °C for 6 h to promote crosslinking reactions. The activated material was then separated by filtration, thoroughly rinsed with distilled water to remove any unreacted glutaraldehyde, and left to dry at room temperature.

After activation, the treated support was immersed in 10 mL of 0.1 M sodium phosphate buffer (pH 7.0) containing 80 units of HRP. Enzyme immobilization was carried out under mild stirring at ambient temperature for 12 h. Following this step, the HRP-functionalized TAC–Cu material was washed multiple times with fresh buffer to remove any non-immobilized enzyme and then dried at room temperature. The protein content was quantified using the Bradford method, with bovine serum albumin serving as the reference protein [20]. The immobilization yield (IY%) was calculated using the following equation:

Immobilization Yield (IY%) = [(Initial protein − Residual protein)/Initial protein] × 100

### 2.4. Horseradish Peroxidase Assay

Horseradish peroxidase (HRP) activity was assessed based on the procedure described by Yuan and Jiang [21]. A 1 mL reaction mixture was prepared, consisting of 40 mM guaiacol, 8 mM hydrogen peroxide (H_2_O_2_), and 50 mM Tris–HCl buffer at pH 7.0. This mixture was combined with either a small quantity of free HRP (20 µL, 4 Units) or a measured amount of HRP immobilized (5 mg) on TAC–Cu. The oxidation of guaiacol was monitored by measuring the increase in absorbance at one-minute intervals. One unit of enzymatic activity was defined as the amount of HRP that caused an increase in absorbance of 1.0 per minute under these specific assay conditions.

### 2.5. Characterization of Material Support

The TAC–Cu composite was thoroughly examined using a suite of analytical tools to verify its successful synthesis and to investigate its structural and morphological characteristics. Crystallinity and phase composition were evaluated through X-ray diffraction (XRD) analysis, performed on a Bruker Advance D8 diffractometer utilizing Cu Kα radiation (λ = 1.5418 Å). Functional groups present in the material were identified using attenuated total reflectance Fourier-transform infrared spectroscopy (ATR-FTIR). ATR-FTIR spectra were recorded using a PerkinElmer Spectrum 100 spectrometer (PerkinElmer Inc., Waltham, MA, USA) over the range 4000–500 cm^−1^ at room temperature. The spectra were collected directly in transmittance mode from the instrument. The raw numerical data were exported as Excel files and replotted using Origin 2018. Because ATR spectra of textile-based samples can be influenced by sample–crystal contact, surface roughness, and sample heterogeneity, the interpretation focused mainly on characteristic band positions and relative spectral changes rather than absolute band intensities. The surface morphology and structural features were visualized using field-emission scanning electron microscopy (FESEM), while the elemental composition and spatial distribution were analyzed via energy-dispersive X-ray spectroscopy (EDX) employing a Bruker Nano XFlash 5010 detector (Bruker Nano GmbH, Berlin, Germany).

To assess the specific surface area and porosity, nitrogen adsorption–desorption isotherms were recorded at 77 K using instrumentation from MicrotracBEL Corp (MicrotracBEL Corp., Osaka, Japan)., supported by Belsorp software (version 6.3.2.1, BEL Japan, Inc.) (MicrotracBEL Corp., Osaka, Japan). The Brunauer–Emmett–Teller (BET) method was used for surface area determination, while the Barrett–Joyner–Halenda (BJH) method was applied to derive pore size distribution from adsorption data. Pore volume was estimated using the density functional theory (DFT) model, specifically employing NLDFT/GCMC analysis. Lastly, surface charge characteristics were determined via zeta potential measurements conducted using a Malvern Zetasizer (version 7.12, (Malvern Panalytical Ltd., Malvern, UK)).

### 2.6. Reusability and Storage Stability of HRP@TAC–Cu

The operational durability of the immobilized HRP was examined by evaluating its reusability over successive catalytic cycles. Following each reaction, the enzyme-bound material was separated from the reaction medium and thoroughly washed with 50 mM sodium acetate buffer (pH 7) to remove residual substrates. The remaining enzymatic activity was quantified and reported as a percentage of the initial activity, allowing assessment of its performance across multiple reuses.

Furthermore, the storage stability of both free and immobilized HRP was investigated over a period of 60 days. Samples were kept at 4 °C, and enzymatic activity was monitored weekly to track retention over time. All activity measurements were normalized to the highest activity recorded on day zero, which was considered 100%, and subsequent values were expressed relative to this reference point.

### 2.7. Kinetic Parameters of Free and Immobilized HRP

The catalytic behavior of both free and immobilized HRP was examined under identical assay conditions using guaiacol as the substrate. The reaction was carried out in 50 mM Tris–HCl buffer (pH 7.0, 50 mM) at 25 ± 2 °C, with a fixed H_2_O_2_ concentration of 8 mM. Guaiacol concentration varied from 40 to 100 mM, while all other assay conditions were kept constant. The oxidation of guaiacol was monitored spectrophotometrically at 470 nm. The resulting initial reaction rates were analyzed using Lineweaver–Burk plots, a double-reciprocal representation of the Michaelis–Menten equation, to determine the kinetic constants *Km* and *Vmax*.

### 2.8. Bisphenol A (BPA) Degradation

The catalytic degradation of bisphenol A (BPA) by free HRP and immobilized HRP@TAC–Cu composites was evaluated using a modified phenolic oxidation method. All experiments were performed in a total reaction volume of 10 mL. For each assay, 10 mL of BPA working solution prepared in sodium phosphate buffer (0.1 M, pH 7.0) was transferred into a 20 mL glass vial, followed by the addition of 50 mg of the immobilized catalyst (plant-based or chemically synthesized) or an equivalent activity of free HRP. The suspension was pre-incubated for 10 min under gentle agitation (100 rpm) to establish adsorption–desorption equilibrium between BPA and the catalyst surface. The reaction was initiated by adding hydrogen peroxide to obtain a final concentration of 1 mM H_2_O_2_. The mixtures were incubated at 25 °C, and 300 µL aliquots were withdrawn at predetermined time intervals (15, 30, 45, 60, 75, and 90 min). To terminate the reaction, 10 µL of sodium azide (0.01 M) was added to inhibit further enzymatic activity.

Residual BPA was quantified spectrophotometrically using the 4-aminoantipyrine (4-AAP)/potassium ferricyanide colorimetric method. Each sample (300 µL) was mixed with 700 µL of phosphate buffer (0.1 M, pH 8.0), followed by the sequential addition of 10 µL of 0.1 M 4-AAP and 10 µL of 0.2 M potassium ferricyanide. After incubation for 15 min at 25 °C, absorbance was measured at 506 nm using a UV–Vis spectrophotometer. A calibration curve prepared from BPA standards (0–0.3 mM) was used to determine the residual BPA concentration. BPA degradation efficiency (%) was calculated according to the following equation:

BPA Removal (%) = [(C_0_ − C_1_)/C_0_] × 100

where C_0_ is the initial concentration of the untreated BPA control and C_1_ is the concentration after treatment.

## 3. Results and Discussion

**Scheme 1:**
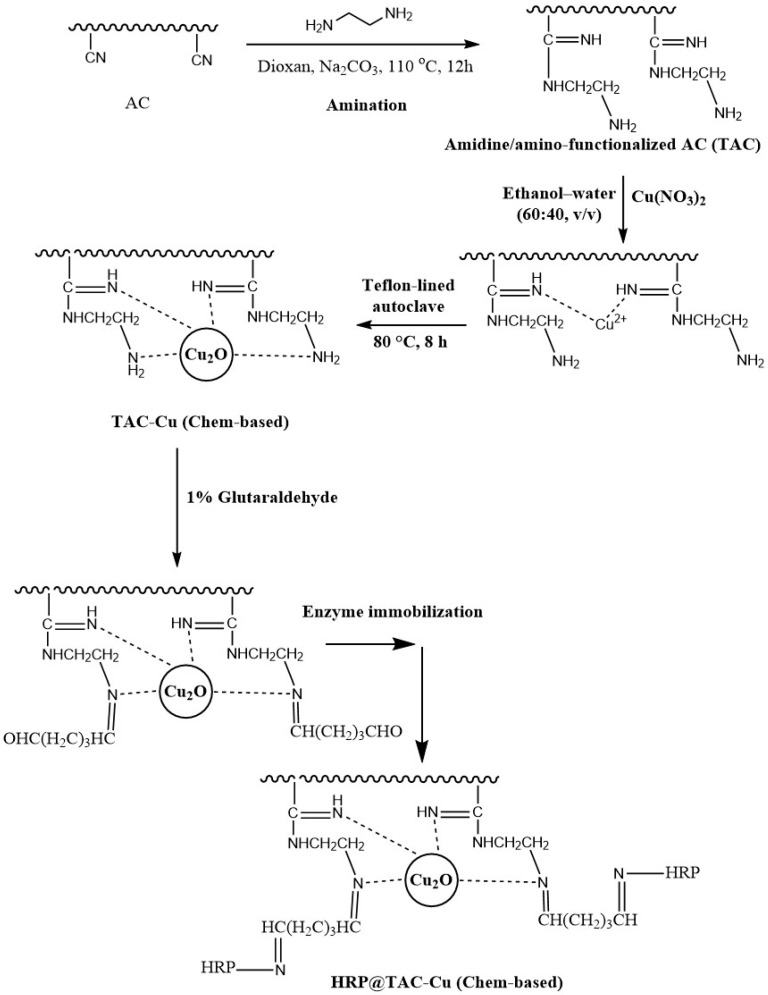
Proposed mechanism for the preparation of chem-based TAC–Cu and covalent immobilization of HRP.

**Scheme 2:**
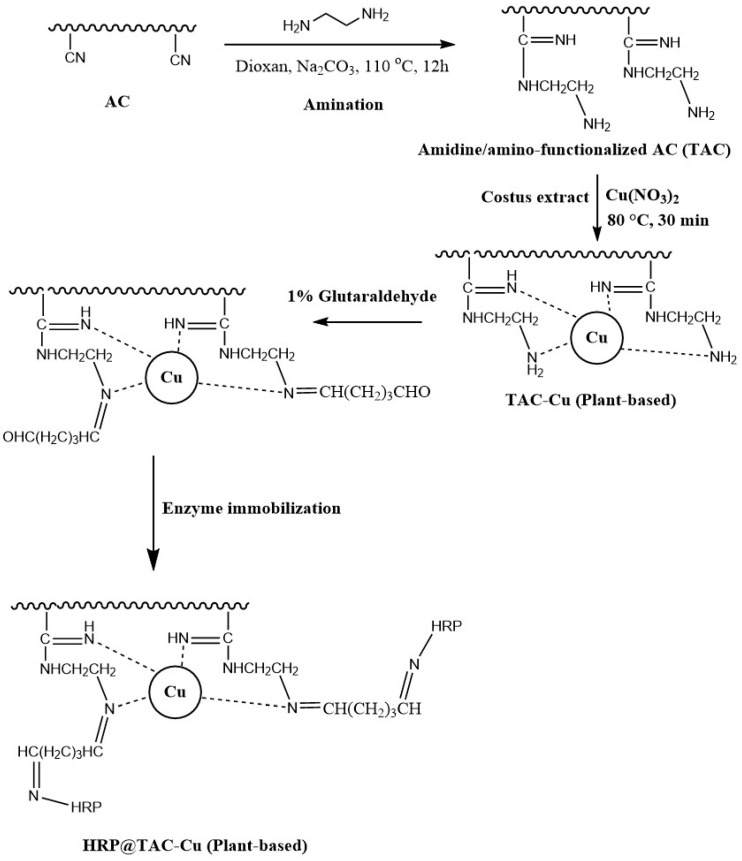
Proposed mechanism for the preparation of Plant-based TAC–Cu and covalent immobilization of HRP.

TAC–Cu represents a novel copper-containing textile hybrid support formed through the interaction of copper species with aminated/amidine-functionalized acrylic textile via either a plant-mediated or a conventional chemical synthesis route. This approach offers a sustainable and potentially biocompatible platform for enzyme immobilization. The uniqueness of the system lies in its dual fabrication pathway, which generates functionalized supports with abundant nitrogen-containing interaction sites and enhanced surface properties, thereby promoting stable enzyme attachment and efficient catalytic performance. The formation of TAC–Cu can be rationalized through sequential surface functionalization, copper species with nitrogen-containing groups, and route-dependent phase formation. First, the nitrile groups of the acrylic textile undergo ethylenediamine-assisted modification, introducing amidine/amino groups onto the TAC surface. Second, Cu^2+^ ions derived from copper nitrate interact with the nitrogen-containing functional groups of TAC. Under solvothermal conditions, these copper species evolve into distinct crystalline phases depending on the synthesis route. In the chemically synthesized sample, the absence of plant-derived reducing and stabilizing phytochemicals favors the formation of cuprite-type Cu_2_O nanoparticles. In contrast, in the plant-mediated route, phytochemicals present in Costus extract likely act as reducing and capping agents, promoting the formation of metallic Cu^0^ nanoparticles with an FCC structure (Scheme 1 and Scheme 2).

In this study, the immobilization yield (IY%) of HRP on TAC–Cu material was assessed for both the plant-based and chemically synthesized versions, resulting in yields of 92% and 84%, respectively. These findings indicate highly effective enzyme immobilization, with the plant-derived support showing better performance. HRP immobilization on TAC–Cu occurs mainly through glutaraldehyde-mediated covalent coupling. During activation, one aldehyde group of glutaraldehyde reacts with surface amino groups on TAC–Cu to form an imine linkage, leaving the second aldehyde group available for reaction with amino groups on HRP. Consequently, HRP is immobilized through Schiff base linkages of the type TAC–N=CH–(CH_2_)_3_–CH=N–HRP. The Cu-based domains contribute mainly to surface roughness, porosity, and interfacial stabilization, while the primary covalent attachment of HRP is attributed to glutaraldehyde bridging between surface amino groups and enzyme amino residues. When compared with previously reported systems, the IY% achieved here is noticeably higher. For example, a functionalized acrylic textile combined with magnetite nanoparticles (TAC–Fe_3_O_4_@β-glucosidase) achieved an IY% of 89% for β-glucosidase [22], which, although high, remains lower than the 91% reported in this work. Systems using silver- or gold-coated amidoximated fabrics for HRP immobilization showed good enzymatic activity and stability, but immobilization yields were not explicitly reported; available information suggests slightly lower performance than the plant-mediated copper-based hybrid system developed here [23]. Additionally, amidrazone-functionalized acrylic fabrics used for α-amylase immobilization reached a maximum yield of 81% [24], further supporting the superior enzyme-loading capability of the TAC–Cu composite in this study. Together, these comparisons highlight the advantages of plant-derived copper-based hybrid structures in improving enzyme immobilization, likely due to their diverse functional groups and biocompatible matrix, which help promote covalent binding and stabilize the enzyme’s structure.

### 3.1. Textural Properties and Pore Structure Analysis

**Figure 1:**
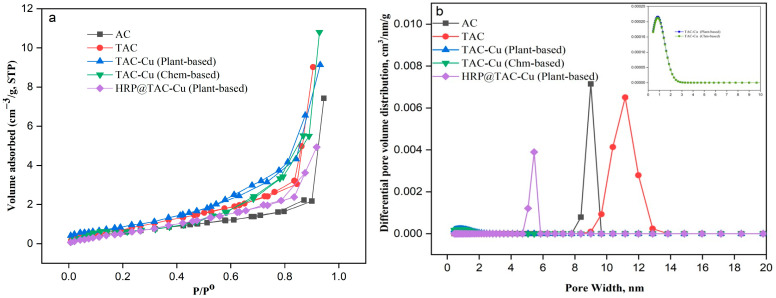
Nitrogen adsorption–desorption isotherms (**a**) and BJH pore size distributions (**b**) of AC, TAC, TAC–Cu, and HRP@TAC–Cu.

The structural evolution of the acrylic fabric after chemical modification and subsequent formation of the copper-based textile hybrid layer was investigated using nitrogen adsorption–desorption isotherms (Figure 1a), BET surface area analysis, BJH pore size distributions (Table 1), and NLDFT pore size profiles (Figure 1b). All samples displayed Type IV isotherms with H3 hysteresis at high relative pressures (P/P_0_ > 0.8), characteristic of mesoporous materials with slit-shaped pores. This mesoporosity was preserved in both AC and TAC, consistent with previous observations in polymer-based coordination hybrids and related porous composite systems [25,26]. The unmodified AC possessed minimal textural development (S_BET_ = 2.03 m^2^/g; BJH pore diameter = 25.03 nm; DFT pore size = 11.15 nm; pore volume = 0.00793 cm^3^/g). Conversion to TAC via ethylenediamine functionalization resulted in a modest increase in surface area (3.86 m^2^/g), a reduction in pore size (16.39 nm BJH; 8.99 nm DFT), and an expanded pore volume (0.0147 cm^3^/g). These changes indicate that ethylenediamine-assisted functionalization introduces reactive functional groups while creating additional accessible porosity—a trend reported in related polymer modification studies aimed at enhancing enzyme immobilization [27]. Formation of the copper-based hybrid structures on the textile surface further refined the material’s textural features. The plant-mediated TAC–Cu composite exhibited the highest surface area (4.47 m^2^/g) and a markedly narrowed pore size (14.27 nm BJH; 0.44 nm DFT), outperforming the chemically synthesized analogue (3.37 m^2^/g; 21.8 nm BJH; 0.44 nm DFT). The DFT-derived microporosity suggests a more compact and uniform framework, likely driven by phytochemicals that act as chelating and templating agents during assembly—an effect noted in green PMCHM syntheses by Grape et al. [28] and Yuliarto et al. [29]. Following enzyme loading, the HRP@TAC–Cu composite retained substantial surface area (3.29 m^2^/g) and showed an increase in pore volume (0.00510 cm^3^/g). Notably, the DFT pore size expanded to 5.44 nm, while the BJH diameter remained unchanged (14.27 nm). This divergence suggests enzyme-induced swelling or internal restructuring within the porous copper-based hybrid structure, in line with confinement-related reorganization reported for porous enzyme-support systems [30,31]. Collectively, these results indicate that the plant-mediated formation of the copper-based hybrid domains on the textile surface improves porosity, structural uniformity, and post-immobilization adaptability. Incorporating DFT analysis provides an accurate view of the microporous architecture, particularly where BJH data alone may obscure true pore dimensions. These findings align with emerging evidence that green synthesis routes not only reduce environmental burden but also yield high-performance supports optimized for biocatalysis and enzyme immobilization [32,33].

Zeta potential measurements further elucidated surface charge behaviour (Table 1). AC exhibited a strongly negative potential (–20.42 mV), reflecting its hydrophobic and unfunctionalized surface. Following ethylenediamine-assisted functionalization, TAC shifted toward neutrality (–0.08 mV), consistent with the introduction of polar amino and amidine-type nitrogen-containing functionalities that enhance surface hydrophilicity. Upon formation of the copper-based textile hybrid structure, the surface charge became distinctly positive, with the plant-derived TAC–Cu material displaying the highest value (+33.52 mV). This pronounced cationic character likely arises from protonated amines, coordinated metal centers, and bio-derived ligands. In contrast, the chemically synthesized analogue exhibited a lower positive potential (+12.2 mV), suggesting reduced surface functionality or less effective metal–ligand coordination. After enzyme immobilization, the zeta potential of HRP@TAC–Cu decreased to +17 mV, likely due to partial charge masking by the protein backbone and the enzyme’s zwitterionic nature at neutral pH.

### 3.2. Magnetic Properties of TAC–Cu Materials

**Figure 2:**
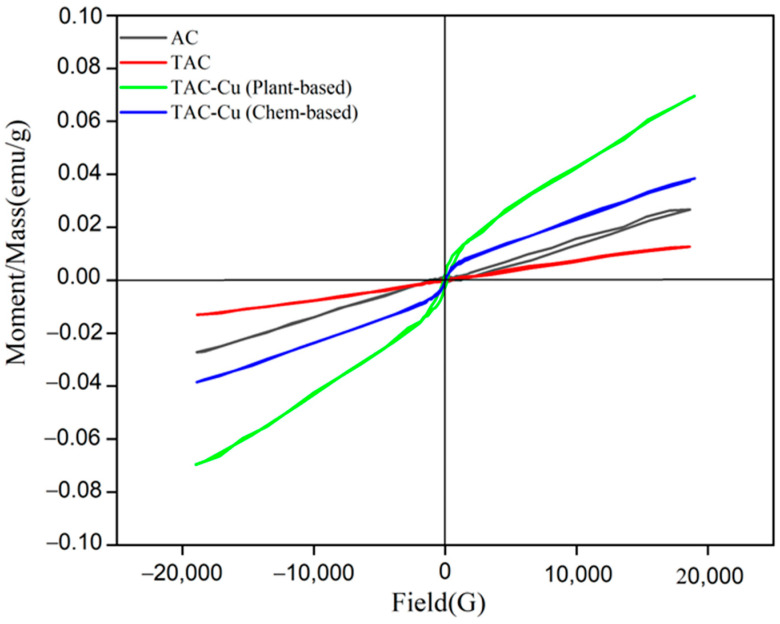
VSM magnetization curves of AC, TAC, and TAC–Cu show variations in magnetic response.

The magnetic behavior of the fabricated materials was evaluated using VSM at room temperature, with the results presented in the hysteresis curves (Figure 2) and summarized in Table 2, which reports the coercivity (*Hc*), saturation magnetization (*Ms*), and remanent magnetization (*Mr*). AC exhibited a very weak magnetic response, with an Ms of 0.0269 emu/g and nearly negligible coercivity (*Hc* = 2.61 G), consistent with its predominantly diamagnetic nature. Following modification to form TAC, a marked increase in coercivity to 345 G was observed, despite a reduction in *Ms* to 0.01284 emu/g. This increase in *Hc* may be associated with the introduction of functional groups and increased surface roughness, which can impede electron spin mobility and impart a weak, paramagnetic-like character. Incorporation of copper into the hybrid structure induced a pronounced change in the magnetic properties. Notably, the plant-mediated TAC–Cu material exhibited an increased saturation magnetization of 0.06956 emu/g and a coercivity (*Hc*) of 140.2 G, indicating the emergence of soft magnetic behavior. This enhancement is attributed to the formation of copper-based nanocrystalline domains within the hybrid structure, which may contribute to a stronger magnetic response. By contrast, the chemically synthesized TAC–Cu sample showed a lower *Ms* of 0.0384 emu/g and near-zero coercivity (*Hc* = 0.036 G), suggesting that the plant-based synthesis route yields a more magnetically responsive and structurally ordered material. The pronounced difference in coercivity between the two variants further suggests that phytochemical-assisted synthesis promotes more uniform formation and dispersion of copper-based domains, leading to enhanced magnetic interactions within the hybrid structure.

Strikingly, after HRP immobilization, the HRP@TAC–Cu (plant-based) sample exhibited a noticeable increase in saturation magnetization (Ms = 0.64 emu/g) and coercivity (Hc = 430.5 G), together with a remanence value of 0.153 emu/g, compared with the corresponding non-immobilized samples. Although the exact origin of this magnetic enhancement cannot be conclusively established from the present data alone, the observed behavior may be associated with interfacial interactions between HRP functional groups and the copper-based hybrid surface, as well as partial structural reorganization within the hybrid matrix after enzyme immobilization. Previous studies have reported that biomolecule loading onto hybrid supports or magnetic nanocomposites can influence magnetic behavior through surface interactions and local structural rearrangements within the inorganic matrix [32,34,35]. However, direct confirmation of possible changes in copper oxidation state or magnetic coupling would require additional analyses such as XPS or EPR spectroscopy.

### 3.3. Crystalline Phase Characterization

**Figure 3:**
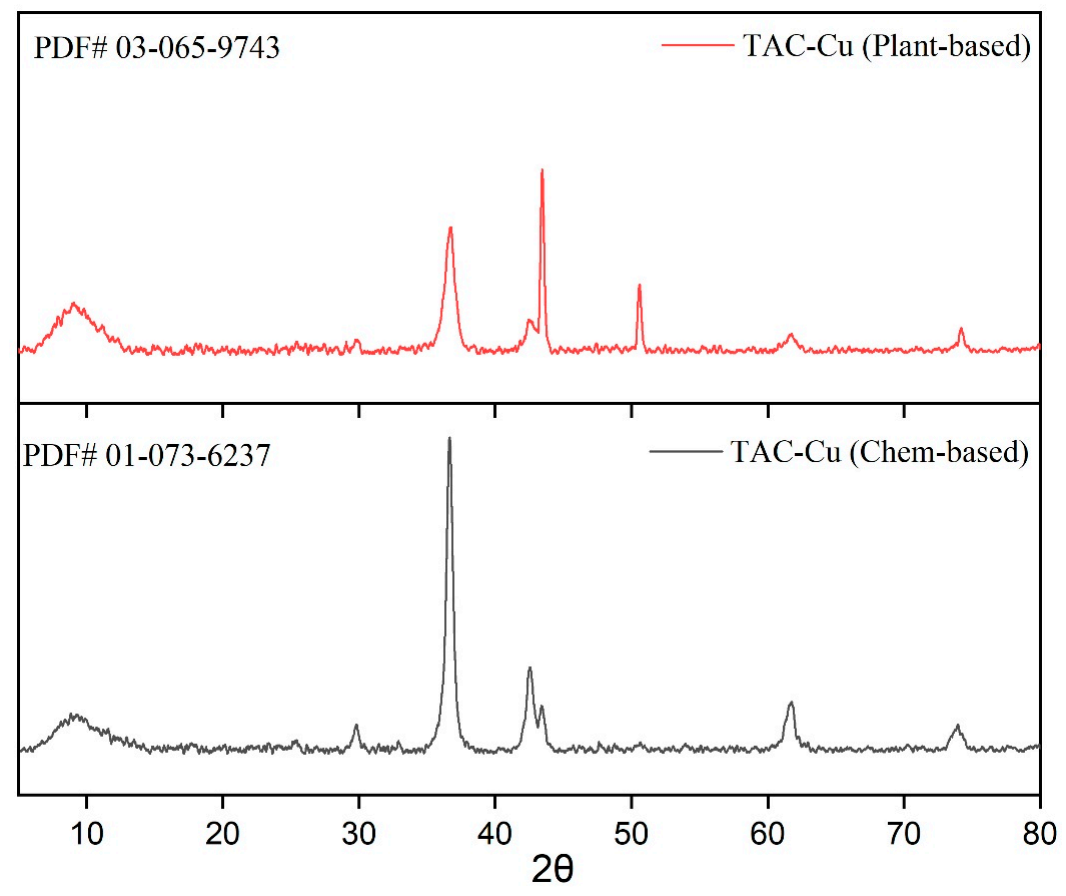
XRD patterns of plant-based and chemically synthesized TAC–Cu materials.

The crystalline structure of the synthesized TAC–Cu composites was investigated using X-ray diffraction (XRD), and the diffraction patterns were indexed against standard crystallographic reference files. As shown in Figure 3, both materials display distinct diffraction peaks corresponding to different cubic phases of copper or copper oxide-based structures. The TAC–Cu (plant-based) material matched PDF# 03-065-9743, assigned to metallic copper with a face-centered cubic (FCC) structure (Fm-3m, space group 225). The major peaks observed at 2θ ≈ 36.7°, 43.4°, 50.6°, 61.7°, and 74.2° correspond to the (111), (200), (220), (311), and (331) planes of cubic Cu, respectively. The relatively sharp and well-defined peaks indicate high crystallinity and uniform grain distribution, likely promoted by plant-derived phytochemicals that act as reducing and capping agents during Cu-coordinated textile hybrid formation. This observation aligns with previous reports showing that phytochemicals facilitate controlled nucleation and restrict crystal growth, yielding more crystalline nanostructures [32,36].

In contrast, the TAC–Cu (chemically synthesized) sample corresponds to PDF# 01-073-6237, assigned to cuprite-type Cu_2_O (Pn-3m, space group 224). The diffraction peaks at 2θ ≈ 36.7°, 42.5°, 43.3°, 61.7°, and 73.9° match the (111), (200), (211), (220), and (311) planes of the cuprite phase. Although these peaks are also sharp, their higher intensity relative to the background suggests the presence of larger crystallites or greater aggregation compared to the plant-based material. The slight differences in peak positions and widths indicate a distinct oxide-based crystalline structure, likely arising from uncontrolled oxidation during synthesis in the absence of reducing phytochemicals. These structural differences have notable implications for the physicochemical behavior of the materials. The metallic copper phase in the plant-based composite can enhance electrical conductivity, catalytic activity, and magnetic responsiveness, whereas the Cu_2_O phase in the chemically synthesized variant is associated with semiconducting properties and comparatively lower catalytic or magnetic efficiency under similar conditions [37]. XRD analysis, therefore, confirms that the green synthesis route promotes the formation of highly crystalline metallic Cu phases, while the conventional chemical method favors oxide-phase formation (Cu_2_O). These findings are consistent with the earlier BET and VSM results, in which the plant-based material exhibited superior textural and functional properties for enzyme immobilization and catalytic applications.

### 3.4. ATR-FTIR Spectroscopic Analysis

**Figure 4:**
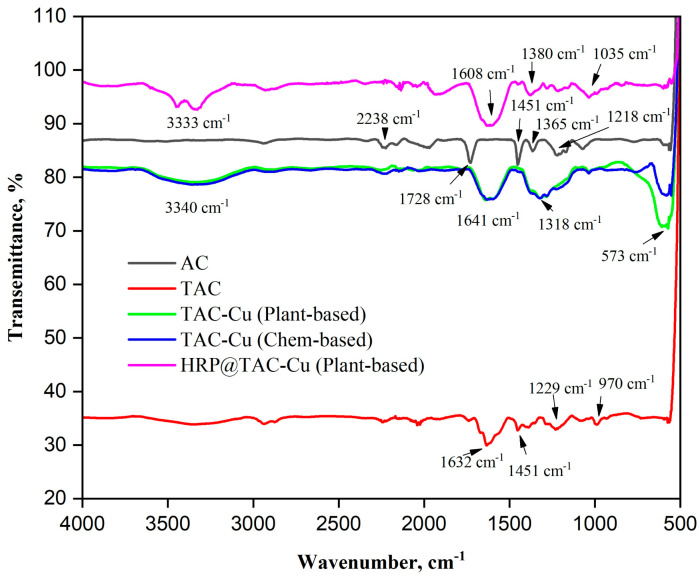
ATR-FTIR spectra of AC, TAC, TAC–Cu, and HRP@TAC–Cu.

The chemical evolution of AC, TAC, TAC–Cu, and HRP@TAC–Cu was evaluated by ATR-FTIR spectroscopy, as shown in Figure 4. The pristine acrylic textile (AC) exhibited characteristic bands associated with commercial acrylonitrile-rich acrylic copolymers. The band at 1728 cm^−1^ is attributed to C=O stretching of ester-containing acrylate/methacrylate comonomer units, while the bands at 1451 and 1365 cm^−1^ correspond to C–H bending vibrations. The band at 1218 cm^−1^ is assigned to C–O stretching. Particular attention was also given to the diagnostic nitrile C≡N stretching region expected for PAN-based acrylic fibers at 2238 cm^−1^. After ethylenediamine treatment, the spectral profile of TAC showed clear changes, including attenuation of the nitrile-related region and the appearance of a new band at 1632 cm^−1^. This band is assigned mainly to C=N stretching associated with amidine-type structures and/or N-containing conjugated groups generated by ethylenediamine-assisted reaction with nitrile groups. The band at 1229 cm^−1^ and the shoulder around 970 cm^−1^ are attributed to C–N stretching and N-containing functional group vibrations. These changes support the introduction of amine/amidine-type functionalities onto the acrylic textile surface [38]. Following copper integration, the TAC–Cu spectra showed additional changes. The plant-based TAC–Cu sample exhibited a broad band around 3340 cm^−1^, corresponding to overlapping O–H/N–H stretching vibrations from surface hydroxyl groups, amine groups, and adsorbed water. The band at 1641 cm^−1^ may arise from overlapping C=N/C=O vibrations of the functionalized textile matrix and plant-derived surface species, while the band at 1318 cm^−1^ is assigned to C–N/C–O stretching vibrations. The low-wavenumber band at 573 cm^−1^ supports the presence of Cu–O and/or Cu–N interactions, consistent with the formation of copper-containing hybrid domains on the functionalized textile surface. Similarly, the chemically synthesized TAC–Cu sample displayed bands around 3340, 1641, and 1320 cm^−1^, together with Cu-related absorptions at 580 cm^−1^, indicating copper incorporation and possible Cu–O/Cu–N interactions. After glutaraldehyde activation and HRP immobilization, the HRP@TAC–Cu spectrum showed further spectral changes consistent with enzyme attachment. The band at 1608 cm^−1^ is attributed to overlapping amide I/C=N vibrations, which may include contributions from protein amide groups and Schiff base linkages formed through glutaraldehyde-mediated coupling between surface amino groups and amino residues of HRP. The bands at 1380 and 1035 cm^−1^ are associated with C–N stretching and amino acid residue-related vibrations. The broad band centered around 3333 cm^−1^ reflects overlapping O–H/N–H stretching from the immobilized enzyme and hydrated hybrid surface.

### 3.5. SEM and EDX Analysis

**Figure 5:**
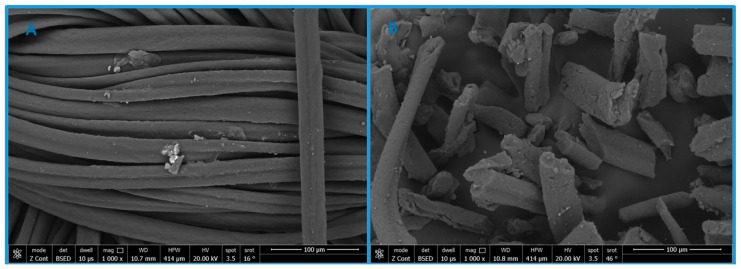

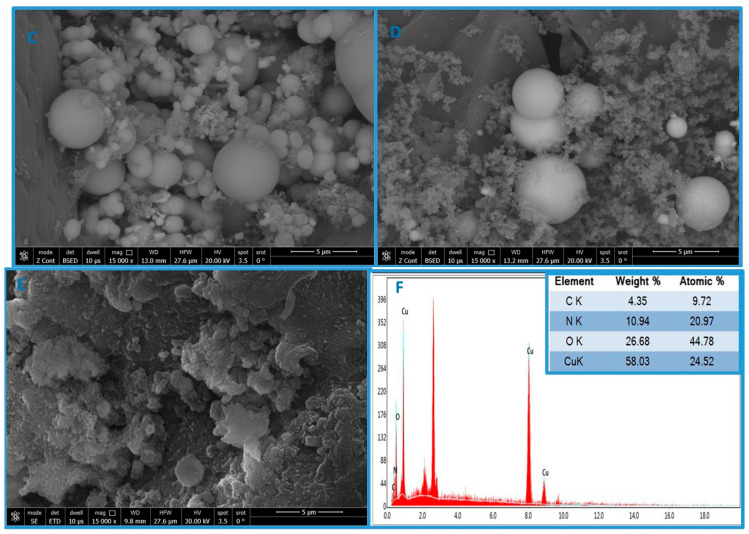
SEM micrographs of (**A**) AC, (**B**) TAC, (**C**) plant-based TAC–Cu, (**D**) chem-based TAC–Cu, and (**E**) plant-based HRP@TAC–Cu, with (**F**) corresponding EDX spectrum showing elemental composition.

The SEM micrographs mainly illustrate the morphological evolution of the acrylic textile during the sequential modification and TAC–Cu formation processes. As shown in Figure 5A, the pristine AC fabric exhibited relatively smooth and well-aligned filaments with limited surface roughness. After ethylenediamine treatment, the TAC sample showed noticeable morphological changes, including increased surface roughness and partial fiber fragmentation (Figure 5B). These changes are consistent with surface modification; however, the introduction of chemical functionalities is primarily supported by ATR-FTIR analysis rather than SEM observation alone.

Following TAC–Cu formation, clear morphological differences were observed between the plant-mediated and chemically synthesized materials. The plant-based TAC–Cu sample displayed densely distributed spherical particles on the textile surface (Figure 5C), suggesting more uniform particle growth and surface coverage, likely assisted by phytochemicals acting as stabilizing and structure-directing agents. In contrast, the chemically synthesized TAC–Cu composite showed a more irregular and aggregated particle morphology (Figure 5D), indicating less controlled nucleation and growth under the chemical synthesis conditions. These morphological observations agree with the BET and XRD results, which showed improved textural features and higher structural uniformity for the plant-derived composite.

After HRP immobilization, the HRP@TAC–Cu (plant-based) sample exhibited a more compact and granular surface texture with partial coverage of the copper-containing particles (Figure 5E). This morphological change may be associated with enzyme immobilization, pore coverage, and surface binding of HRP. The corresponding EDX spectrum (Figure 5F) provides supportive elemental evidence for the presence of Cu, O, N, and C in the hybrid material. The detection of Cu supports the incorporation of copper-containing species into the TAC matrix, while the presence of nitrogen is consistent with nitrogen-rich surface functionalities and the immobilized enzyme. However, EDX analysis was used only as complementary elemental evidence, and the assignment of specific surface functional groups was based mainly on ATR-FTIR characterization. Overall, SEM and EDX results support the morphological and elemental evolution of the material, while ATR-FTIR and XRD provide the principal evidence for chemical functionalization and crystalline phase formation.

### 3.6. Reusability and Storage Stability

**Figure 6:**
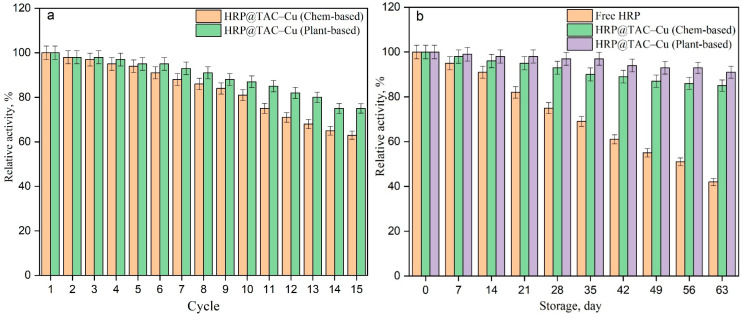
Reusability (**a**) and storage stability (**b**) of free HRP and HRP immobilized on plant-based and chem-based TAC–Cu supports.

The reusability of immobilized enzymes is a critical parameter for their practical application in industrial processes. In this study, HRP was immobilized on TAC–Cu supports synthesized via two different routes: plant-based and chemically based (Figure 6a). The plant-based HRP@TAC–Cu system retained 75% of its initial activity after 15 consecutive reaction cycles, while the chemically based counterpart maintained 61% activity over the same period. These results indicate that both immobilization strategies provide considerable operational stability to HRP, with the plant-based approach offering superior reusability, possibly due to a more favorable microenvironment or reduced structural stress on the enzyme. Compared to prior studies, these findings represent a significant improvement. For example, Zhang et al. reported only 25% residual activity after 7 cycles when HRP was immobilized on graphene oxide [39], and Monier et al. observed 65.8% retention after 6 cycles using modified chitosan beads [40]. Similarly, HRP immobilized on Fe_3_O_4_@PAA-6-arm-PEG-NH_2_ retained 61.06% activity after 8 cycles, and C3-HRP maintained 64% after 10 reuses [41]. Furthermore, HRP immobilized on graphene oxide–SiO_2_ retained 70% activity after 10 cycles, still lower than the 75% observed here [42]. The performance in this study also surpasses that of other immobilized peroxidases, such as SBP on corncob powder and ceramic, which retained only 42% and 63% of their activities after 10 uses [43,44]. The high reusability observed here is attributed to the robust covalent attachment of HRP onto the copper-based textile hybrid structure, which minimizes enzyme leaching and conformational instability. However, the gradual decline in enzymatic activity across cycles may still be attributed to partial denaturation and product accumulation hindering substrate access.

The storage stability of free and immobilized HRP at 4 °C over two months was evaluated to assess the protective effect of immobilization. After 60 days, free HRP retained only 42% of its initial activity, indicating significant deactivation over time, likely due to conformational instability and denaturation under aqueous storage conditions (Figure 6b). In contrast, HRP immobilized on TAC–Cu supports exhibited markedly enhanced storage stability. Specifically, the plant-based TAC–Cu system preserved 91% of initial enzymatic activity, while the chemically synthesized counterpart retained 83%. These results suggest that immobilization provided a protective microenvironment that stabilized the enzyme’s structure, minimizing activity loss during storage. The superior performance of the plant-based system may be attributed to gentler synthesis conditions or improved biocompatibility with the enzyme. When compared to literature, the storage performance of both immobilized systems in this study exceeds that reported by Liu et al., where immobilized HRP retained ~90% activity at 4 °C over 30 days [45], and Vineh et al., where HRP immobilized in PBS retained 74% after 35 days. Notably, the 91% retention over two months in the plant-based system also outperforms the 97% retention observed by Vineh et al. without PBS over 35 days, suggesting long-term stability [46]. The observed deterioration in free HRP aligns with the results of Zhou et al. (2012), who attributed activity loss to structural deformation and disruption of active sites during prolonged storage [47]. Taken together, the results clearly demonstrate that immobilization on TAC–Cu carriers, particularly those derived from plant-based routes, offers a robust strategy for enhancing the long-term stability of HRP under storage conditions.

Although the HRP@TAC–Cu biocatalyst exhibited good operational reusability, the long-term stability of the copper-based support itself requires further investigation. In the present study, copper leaching into the reaction medium after repeated catalytic cycles or under elevated temperature conditions was not quantitatively measured. Therefore, the environmental safety and long-term durability of the support should be interpreted cautiously. Future studies should include direct determination of released Cu species, for example by ICP-OES or ICP-MS, after multiple reuse cycles and thermal exposure to evaluate the structural stability and practical applicability of the material under wastewater treatment conditions.

### 3.7. Kinetic Analysis

**Figure 7:**
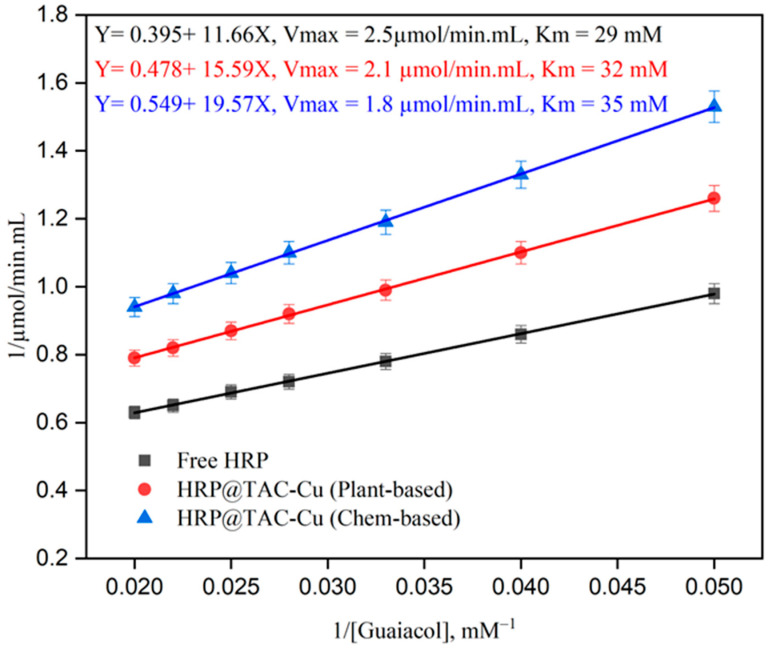
Lineweaver–Burk plots of free HRP and HRP immobilized on plant-based and chem-based TAC–Cu supports.

The kinetic analysis provides critical insight into how immobilization on the Cu hybrid matrices influences the catalytic performance of HRP (Figure 7). As expected, immobilization altered both the substrate affinity and the turnover capacity of the enzyme, consistent with widely observed trends in heterogeneous biocatalysts. Free HRP exhibited the lowest Km value (29 mM), indicating the highest affinity for guaiacol. Following immobilization, Km increased to 32 mM for the plant-based TAC–Cu material and 35 mM for the chemically synthesized variant. This moderate increase in Km is a common consequence of enzyme immobilization, reflecting the mass-transfer limitations and steric hindrance that substrates must overcome to reach the active sites within the support’s microstructure [48]. The substrate must diffuse through the copper-based hybrid layer, thereby introducing an additional diffusion-resistance contribution to the overall reaction rate. The difference between the two immobilized systems is particularly revealing. The plant-based support, with its more uniform morphology, higher zeta potential, and refined microporous network, imposed less diffusion resistance and therefore maintained substrate affinity closer to the native enzyme. This suggests that the biomineralization or de novo approach facilitated by phytochemicals creates a more favorable and less restrictive microenvironment for the enzyme [49]. In contrast, the chemically synthesized composite, characterized by greater aggregation and a less organized pore network, showed the highest Km, indicating a more restrictive microenvironment and greater diffusional constraints.

A decrease in Vmax is commonly observed upon immobilization due to partial conformational restriction and reduced freedom of active-site dynamics [48]. Here, free HRP demonstrated the highest Vmax (2.5 µmol/min. mL), followed by the plant-based immobilized enzyme (2.1 µmol/min. mL) and the chemically synthesized support (1.8 µmol/min. mL). The superior Vmax retention of the plant-based material can be attributed to: (i) better surface dispersion of copper-based hybrid particles facilitated by phytochemicals; (ii) enhanced electron-transfer pathways within the copper-based hybrid structure; and (iii) a more biocompatible microenvironment that limits structural distortion of the enzyme, preserving its intrinsic catalytic efficiency [50]. Meanwhile, the lower Vmax in the chemically synthesized support likely arises from less favorable enzyme orientation and stronger steric confinement, reducing catalytic turnover. Although both immobilized systems exhibit slightly reduced substrate affinity and turnover rates compared to free HRP, the plant-based TAC–Cu composite consistently outperformed the chemically synthesized one. These kinetic improvements align with earlier structural data (BET, DFT pore size, SEM morphology, VSM magnetic behavior), which collectively indicate that the plant-assisted synthesis yields a more homogeneous and catalytically accessible framework. The nearly retained catalytic efficiency, combined with enhanced robustness, makes the plant-based TAC–Cu support an attractive platform for sustained biocatalytic applications, particularly in wastewater treatment and phenolic pollutant degradation.

Although immobilized HRP exhibited slightly lower Vmax values than free HRP in the guaiacol assay, this behavior does not contradict the higher pseudo-first-order degradation rate constants observed during BPA removal. The Vmax values reflect the intrinsic maximum catalytic turnover of HRP toward guaiacol under homogeneous Michaelis–Menten assay conditions, whereas the BPA degradation constant (k) represents the overall performance of the heterogeneous HRP@TAC–Cu system. Immobilization may reduce the apparent Vmax because of diffusional resistance, steric hindrance, and partial restriction of enzyme mobility. However, the TAC–Cu support simultaneously provides several advantages during BPA degradation, including improved enzyme stability, protection against oxidative deactivation, enhanced interfacial interaction between BPA and the catalyst surface, and local substrate enrichment near the immobilized enzyme. These combined effects can lead to improved apparent BPA degradation kinetics despite a modest reduction in intrinsic enzymatic turnover measured using the model guaiacol substrate.

### 3.8. BPA Removal

**Figure 8:**
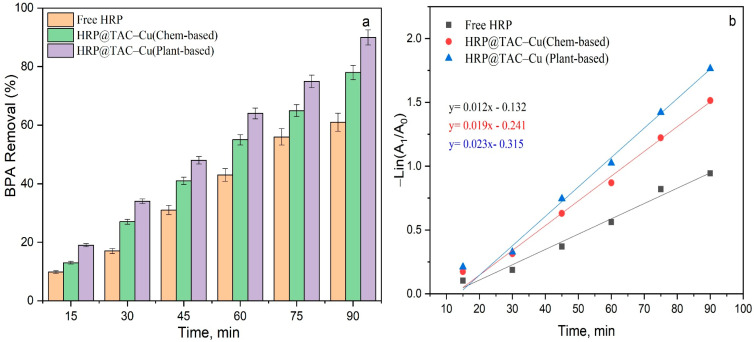
BPA degradation profiles (**a**) and kinetic plots (**b**) for free HRP and HRP immobilized on plant-based and chem-based TAC–Cu supports.

The time profile of BPA degradation using free HRP and the immobilized catalysts is presented in Figure 8a. All systems showed a gradual increase in BPA removal over time; however, the immobilized HRP clearly outperformed the free enzyme throughout the entire reaction period. During the first 30 min, removal efficiencies remained relatively low (17–34%), reflecting the early stages of phenoxy-radical formation and polymerization. As the reaction progressed, significant differences emerged. At 60 min, free HRP achieved 43% removal, while HRP@TAC–Cu (chem-based) and HRP@TAC–Cu (plant-based) reached 55% and 64%, respectively. By 90 min, the performance gap widened considerably. Free HRP removed 61% of BPA, whereas the chemically synthesized HRP@TAC–Cu achieved 78%, and the plant-based HRP@TAC–Cu reached 90%. The markedly higher efficiency of the immobilized systems is attributed to enhanced substrate adsorption, improved electron transfer within the Cu–coordinated framework matrix, and reduced enzyme deactivation over time [45,51]. The superior removal observed for the plant-derived composite further supports its more favorable surface properties and microenvironment for HRP catalysis. The degradation kinetics of BPA were examined using pseudo-first-order kinetics, based on the linear relationship between −ln(A_1_/A_0_) and reaction time (Figure 8b). All three catalytic systems produced highly linear plots, confirming that BPA oxidation proceeds via apparent first-order behavior under the applied conditions. The calculated rate constants (k), derived from the slopes of the fitted lines, were free HRP, k = 0.012 min^−1^; HRP@TAC–Cu (chem-based), k = 0.019 min^−1^; and HRP@TAC–Cu (plant-based), k = 0.023 min^−1^. The immobilized catalysts demonstrated significantly higher reaction rates than free HRP, with the plant-based composite displaying the highest kinetic constant, nearly double that of free HRP. This improvement highlights the benefits of immobilization, including increased substrate accessibility, enhanced microenvironmental stability, and reduced diffusion limitations [52]. Additionally, the presence of copper-based active sites within the porous hybrid structure may promote efficient interfacial electron transfer among HRP, H_2_O_2_, and BPA, thereby enhancing the catalytic cycle [14]. The superior kinetic performance of the plant-derived TAC–Cu material aligns with its enhanced surface properties, higher surface charge, and more uniform structural features, which collectively promote faster and more efficient BPA oxidation.

**Figure 9:**
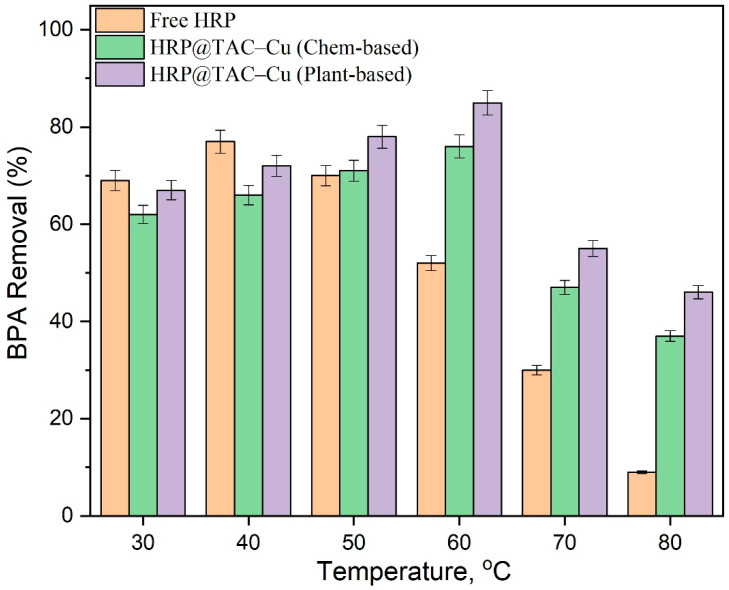
Temperature-dependent BPA removal efficiency of free HRP and immobilized HRP on TAC–Cu supports.

The influence of temperature on BPA degradation by free HRP and the two immobilized biocatalysts is shown in Figure 9. All catalysts exhibited measurable activity across the tested temperature range (30–80 °C), but clear differences in thermal tolerance and catalytic behavior were observed. At 30 °C, BPA removal efficiencies were comparable among the three systems, with free HRP achieving 69%, the chem-based HRP@TAC–Cu reaching 62%, and the plant-based composite showing 67%. As the temperature increased to 40 °C, free HRP displayed its highest performance (77%), consistent with the known optimum for soluble HRP.

A notable shift occurred at 50–60 °C, where the immobilized catalysts significantly outperformed the free enzyme. At 60 °C, BPA removal by the chem-based and plant-based composites reached 76% and 85%, respectively, compared to only 52% for free HRP. These results highlight the enhanced structural stability conferred by the Cu-coordinated framework, which protects the enzyme from thermal unfolding and preserves heme accessibility during catalysis [35]. At higher temperatures (70–80 °C), free HRP exhibited a dramatic loss of activity, dropping to 30% removal at 70 °C and nearly complete deactivation (9%) at 80 °C. In contrast, both immobilized systems retained substantial activity under the same conditions. The plant-based composite again demonstrated superior resilience (55% and 46% removal at 70 °C and 80 °C, respectively). This pronounced thermal stability can be attributed to the rigidification of the enzyme conformation following covalent attachment, together with the protective microenvironment created by the copper-based hybrid matrix, which is particularly advantageous for industrial wastewater treatment [52,53]. Overall, the temperature-dependent behavior confirms that the plant-based TAC–Cu support offers the most robust thermal protection for HRP, maintaining high catalytic performance even at temperatures that completely denature the free enzyme.

## 4. Conclusions

In this study, we successfully developed a novel, hybrid textile-based biocatalyst by immobilizing HRP onto a copper-based hybrid domains anchored on aminated/amidine-functionalized acrylic textile (TAC). The key innovation lies in the use of *Costus speciosus* extract, which facilitated the green synthesis of the plant-based TAC–Cu composite. This biogenic approach yielded a material with superior properties, including a high immobilization yield (91%) and a more favorable microenvironment for HRP. The resulting biocatalyst demonstrated exceptional performance, exhibiting a reaction rate constant for BPA degradation nearly double that of free HRP and achieving an 90% removal efficiency. Crucially, the copper-based hybrid matrix conferred significant operational advantages, including enhanced thermal stability—allowing the enzyme to retain substantial activity at 80 °C—and excellent reusability. This work validates the potential of integrating green chemistry with textile functionalization to create robust, sustainable, and high-performance biocatalytic platforms for industrial wastewater treatment and environmental remediation.

## Figures and Tables

**Table 1:** Textural Properties and Surface Charge Characteristics of AC, TAC, TAC–Cu Composites, and HRP-Immobilized Material.

Samples	S_BET_ (m^2^/g)	Pore Diameter (BJH) (nm)	Total Pore Volume (DFT) (cm^3^/g)	Zeta Potential (mV)
AC	2.03	25.03	0.00793	−20.42
TAC	3.86	16.39	0.0147	−0.08
TAC-Cu (Plant-based)	4.47	14.27	0.00376	33.52
TAC-Cu (Chem-based)	3.37	21.8	0.00371	12.2
HRP@TAC-Cu (Plant-based)	3.29	14.27	0.00510	17

**Table 2:** Magnetic Properties of AC, TAC, Cu Hybrids, and HRP-Immobilized TAC–Cu Composites.

	Coercivity (*Hc*) (G)	Saturation Magnetization (*Ms*) (emu/g)	Remanent Magnetization (*M*r) (emu/g)
AC	2.61	0.0269	866 × 10^−6^
TAC	345	0.0128	162 × 10^−6^
TAC-Cu (Plant-based)	140.2	0.0695	2.58 × 10^−3^
TAC-Cu (Chem-based)	0.036	0.0384	2.77 × 10^−6^
HRP@TAC-Cu (Plant-based)	430.5	0.64	0.153

## Data Availability

The original contributions presented in this study are included in the article. Further inquiries can be directed to the corresponding authors.
